# Successful Laparoscopy-Assisted Extirpation of Burkitt’s Lymphoma Causing Intestinal Obstruction in a 17-Year-Old Boy

**DOI:** 10.3390/jcm13247834

**Published:** 2024-12-22

**Authors:** Zoltán Derzsi, Zsolt Bara, Emőke Horváth, Gabriel Serac, Răzvan Mărginean, Réka Sólyom, Evelyn Kovács, Horea Gozar

**Affiliations:** 1 Department of Pediatric Surgery and Orthopedics, George Emil Palade University of Medicine, Pharmacy, Science and Technology of Targu Mures, 540142 Târgu Mureș, Romania; zoltan.derzsi@umfst.ro (Z.D.); horea.gozar@umfst.ro (H.G.); 2 Clinic of Pediatric Surgery and Orthopedics, Targu Mures, County Emergency Clinical Hospital, 540136 Târgu Mureș, Romania; 3 Pathology Service, Târgu Mureș, County Emergency Clinical Hospital, 540136 Târgu Mureș, Romania; emoke.horvath@umfst.ro; 4 Department of Pathology, George Emil Palade University of Medicine, Pharmacy, Science and Technology of Targu Mures, 540142 Târgu Mureș, Romania; 5 Department of Surgery, George Emil Palade University of Medicine, Pharmacy, Science and Technology of Targu Mures, 540142 Târgu Mureș, Romania; gabriel.serac@umfst.ro; 6 Department of Pediatrics, George Emil Palade University of Medicine, Pharmacy, Science and Technology of Targu Mures, 540142 Târgu Mureș, Romania; reka.solyom@umfst.ro

**Keywords:** Burkitt lymphoma, laparoscopy, children, intestinal obstruction, intussusception

## Abstract

**Background:** Childhood extranodal B-cell non-Hodgkin’s lymphomas are often caused by Burkitt’s lymphoma (BL). Treatment usually involves intensive polychemotherapy, and recent prospective trials show significantly improved outcomes. Surgery primarily involves conducting biopsies; ablative interventions are not recommended. However, in cases of severe presentation, such as an acute abdomen, emergency surgery may be necessary. **Methods:** We present the case of a 17-year-old boy who underwent emergency surgery due to intestinal obstruction caused by a tumor mass. An exploratory laparoscopy was performed due to abdominal wall infiltrates, and a large intraabdominal mass was discovered in the ileocaecal region. The tumor and tumor infiltrates were successfully removed en bloc in a minimally invasive laparoscopy-assisted fashion. **Results:** The postoperative course was favorable, and chemotherapy was started. Histopathology confirmed the diagnosis of BL. Follow-up examinations, including a positron emission tomography (PET) scan, showed no tumor recurrence. More than two years later, the patient remains asymptomatic with negative PET scans. **Conclusions:** Laparoscopy-assisted removal can be useful for pediatric solid abdominal tumors with abdominal wall infiltrates that cause obstruction. Surgeons must assess indications and procedures based on imaging and findings during emergency interventions. The role of ablative MIS in pediatric BL is limited.

## 1. Introduction

In children, Burkitt’s lymphoma (BL) is a common subtype, comprising 40% of B-cell non-Hodgkin’s lymphomas (B-NHLs). It is an aggressive and rapidly growing tumor of lymphoid tissue with extranodal involvement in more than 80% of cases. It usually develops in the gastrointestinal tract, head, and neck and is often diagnosed at an advanced stage [1,2]. It causes different clinical and radiologic presentations. In sporadic forms, abdominal involvement is common [3]. The incidence of BL worldwide is increasing, especially among older male children. Eastern Europe is estimated to have 1.9 to 2.9 cases per million [3].

Of all childhood tumors, BL is the most likely to cause complications [4]. Regarding abdominal localization, the tumor primarily affects the end of the ileum, cecum, and appendix due to the abundance of lymphoid tissue in the ileocecal junction. Intussusception is a possible complication in 18% of abdominal BLs, and the incidence increases with age [5].

The main treatment for BL is chemotherapy based on regimens such as the Berlin–Frankfurt–Münster (BFM) protocol. Surgery may only be needed to obtain biopsy material for a prompt diagnosis. Curative tumor ablation is not considered a treatment option. Delaying effective chemotherapy can be dangerous and carries a high risk of complications. Unfortunately, an acute abdomen may develop, making emergency interventions necessary. Following chemotherapy plans, “Second-look operations” may be required to identify metastatic tissues [6]. Firstly, for children, Holcomb et al. proposed oncological indications for laparoscopy and minimally invasive surgery (MIS) based on data from the Children’s Cancer Group. These indications included biopsies, staging, and determination of resectability [7].

In acute abdomen cases, complete remission after surgical excision alone has been reported in a very early stage of the disease [8]. Adequate excision is required if complete tumor removal is attempted, followed by bowel anastomosis [9]. After that, the treatment plan must include 2 to 6 months of intensive pulse polychemotherapy. Toxicity remains a challenge in many patients.

Recurrence tends to occur within the first year; however, several consecutive prospective trials have shown a significant improvement in outcomes [1,10]. The five-year overall survival rate has increased from 70% in the early 1990s to almost 90% in numerous developed countries [11].

## 2. Case Report

The family history revealed no instances of lymphomas or neoplasia. One year prior to the tumor’s detection, the boy was taken to the emergency department (ED) due to rectal and abdominal pain accompanied by bilious vomiting. After admission to the pediatric surgery ward, the patient was transferred to the operating room, and emergency surgery was performed with a median laparotomy. An ileocecal intussusception was decelerated. Disinvagination, appendectomy, and lavage were performed. Despite local edema, no pathological lead point was identified during the procedure. Except for the appendix, no biopsies were made. The specimen did not show any pathological changes. The recovery was favorable.

**Figure 1:**
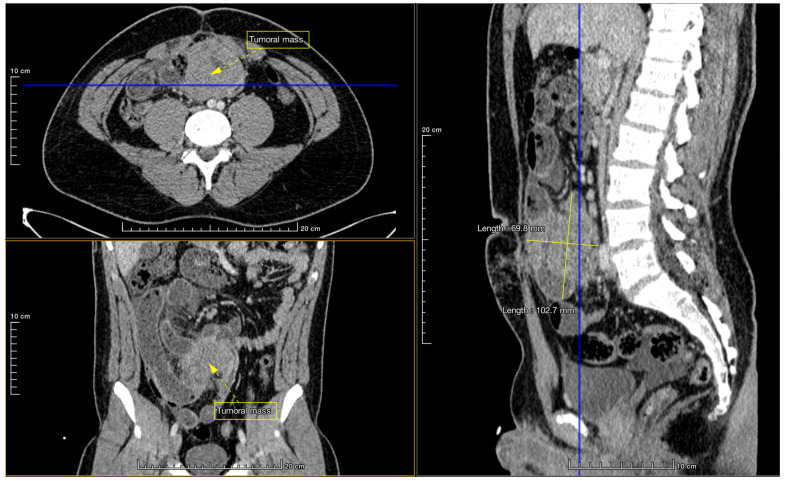
Preoperative CT scan: A loop of the ileum shows a thickened wall (tumoral mass) about 70 mm thick over a length of 10 cm, with findings associated with intravenous contrast media throughout the parietal volume. There is almost total stenosis of the lumen, possibly linked to nodular lymphoid hyperplasia, intestinal intussusception, or intestinal lymphoma, leading to intestinal occlusion. Multiple hydroaeric levels were observed in the small intestine, and enlarged mesenteric lymph nodes measuring up to 18 mm in diameter were present.

One year later, the 17-year-old patient started again with colicky abdominal pain; he was brought to the ED again. He had no associated symptoms, nausea, or vomiting. He had felt similar but moderate abdominal pain a month before, which disappeared spontaneously. An examination revealed abdominal tenderness, primarily in the lower quadrants, and a painful palpable mass on the right iliac fossa. Abdominal ultrasound (US) and computer tomography (CT) scans were performed. CT scan revealed a contrast-absorbing ileocecal tumoral mass (thickening of an ileal loop) infiltrating the abdominal wall, which obstructed the lumen. Multiple enlarged mesenteric lymph nodes were also seen. Additionally, air-fluid levels were present, and peritoneal, perihepatic, and interileal fluid was described (Figure 1).

The boy was admitted to the pediatric surgery ward. His state could not permit other investigations, such as complete blood count (CBC), coagulation tests, and lactate dehydrogenase (LDH) level measuring. CBC showed minor and non-relevant modifications. LDH was moderately increased by 251 U/L. Emergency surgery was unavoidable due to the acute abdomen.

**Figure 2:**
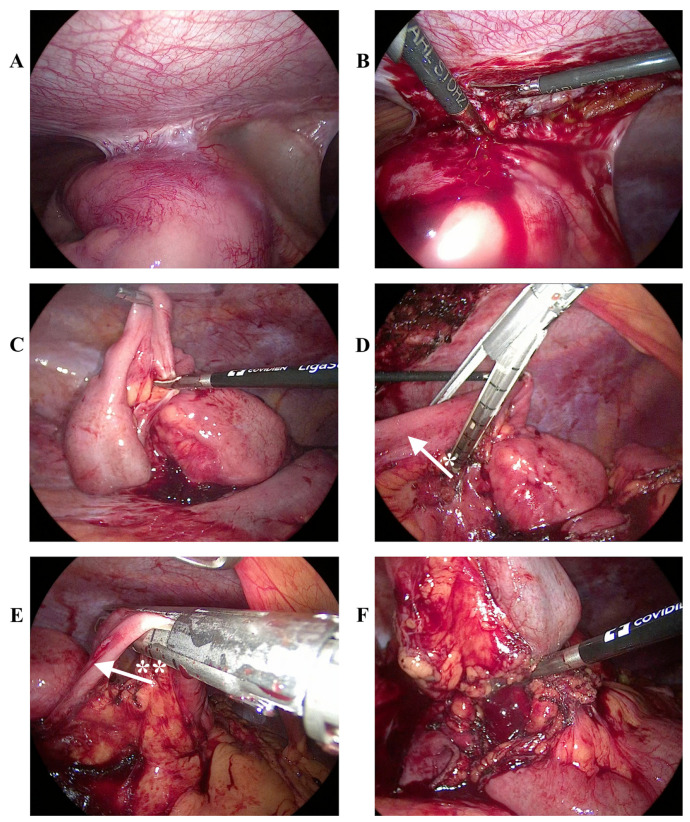
Intraoperative laparoscopic images: (**A**) anterior abdominal wall infiltrates; (**B**) dissection of the adhesions; (**C**) division of the mesentery of the adjacent ileocaecal loop with Medtronic LigaSure™ device; (**D**,**E**) intestinal resection using two 12 mm Covidien Medtronic Endo GIA™ Universal Staplers; (**F**) extirpation of the tumoral basement. *: afferent bowel loop; **: efferent bowel loop.

After a rapid analysis of the images, the surgical team opted for minimally invasive laparoscopic access to better approach the midline anterior abdominal wall infiltrates. This decision was aimed at enhancing patient recovery and reducing postoperative complications. Minimally invasive techniques create smaller incisions, leading to less pain, quicker healing times, and shorter hospital stays. The team believed this approach would provide better visualization of and access to the affected areas, thereby improving surgical outcomes. The use of laparoscopic tools and techniques also helps minimize tissue damage and ensure a more precise intervention. Trocar placement was carried out based on the CT scan: a 12 mm trocar was placed on the left anterior axillary line above the level of the umbilicus, and 10 mm and 5 mm ports were placed on the median left axillary line, maintaining triangulation. A large intraabdominal mass infiltrating the abdominal wall was identified in the ileocaecal region. After careful dissection of the tumor from the peritoneal adhesions using a LigaSure™ (Covidien, Mansfield, MA, USA) device, the mesentery of the adjacent ileocaecal loop was divided. En bloc resection of the tumor and peritoneal infiltrates was achieved. Intestinal resection was also performed using two 12 mm Endo GIA™ Universal Staplers (Covidien, Mansfield, MA, USA). The tumor was removed by a minimal sub-umbilical laparotomy. Latero-lateral mechanical suture anastomosis of the ileal bowels was carried out with an Open Linear Stapler (Covidien, Mansfield, MA, USA). A peritoneal drainage catheter was inserted. All specimens were sent for histopathological studies. Figure 2 shows the intraoperative laparoscopic images.

After the intervention, the patient’s recovery was favorable. their bowel movements resumed on the 3rd postoperative day.

Histopathological examination of the excised bowel fragment (Figure 2A) showed an atypical diffuse lymphoid proliferation with a relatively monomorphic appearance, circumferentially involving the entire wall thickness, causing mucosal ulceration and extending into the mesentery with multiple vascular invasions, without the involvement of the resection margins and peritumoral lymph nodes. Tumor proliferation was represented by medium-sized tumor cells with rough chromatin nuclei and multiple small prominent nucleoli, as well as frequent mitosis. Numerous apoptotic bodies and macrophages with cytoplasmic debris were observed among the tumor cells, giving a “starry sky” appearance (Figure 2B). To establish a definitive diagnosis, a broad panel of antibodies was used to characterize the immunophenotype of the tumor (Table 1).

**Figure 3:**
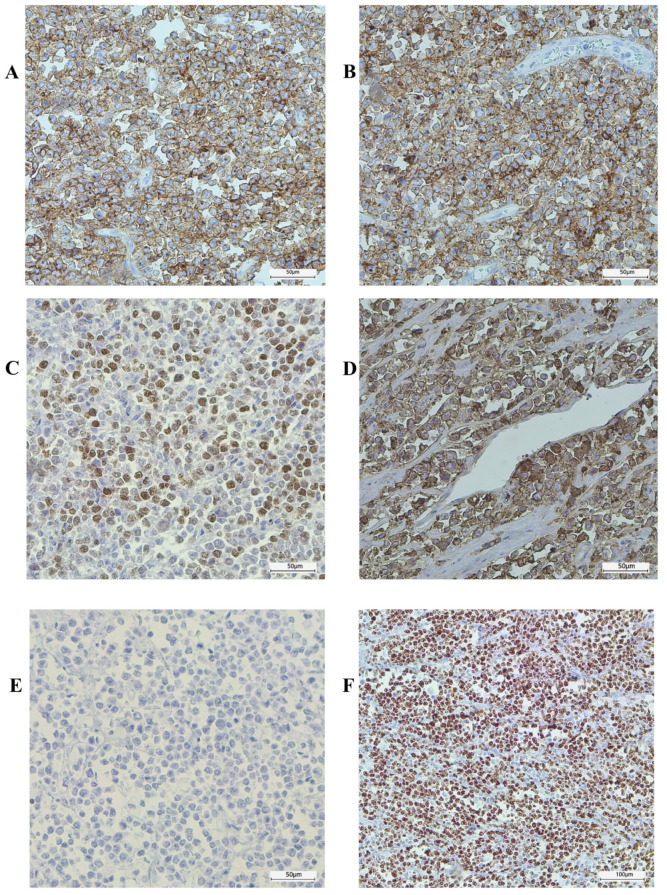
Tumor immunohistochemistry (DAB chromogen). The tumor cells showed a characteristic immunophenotype, being positive for CD20 (**A**) and CD10 (**B**), BCL-6 (**C**), and immunoglobulin M (**D**) without BCL-2 expression (**E**). A high proliferation rate was observed, with a Ki67 proliferation fraction approaching 100% (**F**).

**Figure 4:**
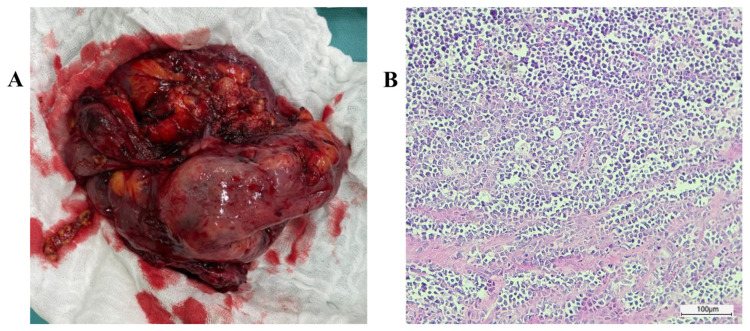
Large ileocecal tumor mass with mesenteric involvement (**A**) histologically suggestive of Burkitt’s lymphoma, consisting of a monomorphic proliferation of intermediate-size cells with a “starry sky” appearance; H&E stain (**B**).

Through immunohistochemistry, the tumor cells were found to express B-cell markers, including CD20 (Figure 3A) and PAX5, and co-express the germinal center markers CD10, BCL6, and IgM (Figure 3B–D), and they were found to be negative for BCL2 (Figure 3E), Cyclin D1, CD23, CD5, and TdT. The Ki67 proliferation rate was close to 100% (Figure 3F). CD3 and CD5 reactions only detected T lymphocytes in very low numbers among the tumor cells. Epstein–Barr virus (EBV) and c-Myc immunohistochemistry were also performed, but for technical reasons, the results were uninterpretable. Although we were not able to prove c-Myc rearrangement, based on the histological appearance and immunophenotype of the tumor cells, a diagnosis of Burkitt’s lymphoma of the terminal ileum was made, supported by the patient’s age and the location of the tumor mass. Figure 3 and Figure 4 show the specimen and the histopathologic images.

A multidisciplinary team composed of pediatric hemato-oncologists, pediatric surgeons, adult surgeons, and anesthesiologists came together to discuss the case. This team allowed for a comprehensive approach to the patient’s care. Their collaboration enhanced their ability to effectively address complex medical issues and ensured that the treatment plan was well rounded and considered all aspects of the patient’s health and treatment needs.

On the 7th postop day, the patient was transferred to a pediatric oncology ward for further therapy and investigations. A positron emission tomography (PET) scan was carried out before initializing chemotherapy. Besides two enlarged retroperitoneal fluorodeoxyglucose (FDG) non-capturing lymph nodes and artifacts caused by the surgical intervention, no other modifications were identified. The tumor was graded as stage 2 BL. The chemotherapy regimen recommended by the non-Hodgkin lymphoma Berlin–Frankfurt–Muenster study group (B-NHL BFM 2004 trial) was started with intrathecal methotrexate (MTX), Cytosar, and dexamethasone, followed by cycle A—vincristine, MTX, ifosfamide, dexamethasone, and Cytosar—and cycle B—vincristine, MTX, and cyclophosphamide—for two months. After two years of follow-up, the patient was asymptomatic. Since then, regularly effectuated PET scans and analyses have been negative for tumor relapses.

## 3. Discussion

BL, the most common childhood lymphoma, is a subtype of highly aggressive B-cell lymphoma with well-characterized clinicopathological and molecular features. The 2022 WHO classification [12] recognizes three forms of BL: endemic, non-endemic or sporadic, and immunodeficiency-associated. However, these traditional subtypes have lost their relevance in understanding the role of EBV in disease pathogenesis [13,14]. Current data show that EBV-positive and EBV-negative BLs form distinct biological groups based on their molecular characteristics [12]. A hallmark of these lymphomas is the chromosomal translocation of the c-Myc oncogene and immunoglobulin locus regulatory elements (t(8;14)) between c-Myc (8q24) and the immunoglobulin heavy chain gene on chromosome 14q32 (IGH-14q32) [15], but c-Myc rearrangement is positive in other high-grade lymphomas, such as high-grade B-cell lymphoma (HGBL), not otherwise specified (NOS). These interesting subtypes occur at an older age and are associated with a proliferation rate of typically less than 90% [16]. This is why we did not carry out a translocation test at the time of diagnosis. We also considered that diagnosing Burkitt’s lymphoma is a histopathological emergency because it is the fastest-growing human tumor with a rapid doubling time, which does not allow for diagnostic delay. Elevated LDH and increased uric acid levels are common findings with prognostic relevance [17]. Our patient had a moderately increased level [18], which the acute abdomen state might have also influenced.

Small bowel tumors are characterized by their non-specific symptoms, which allow them to grow and reach advanced stages before detection [19]. Few published studies specifically address emergency surgery for small bowel tumors with a focus on clinical presentation. They are reported to cause surgical emergencies in 25–67% of cases. Many studies indicate a correct preoperative diagnosis in only 21–53% of patients [20]. In a recent systematic review by Butterworth et al. on an acute abdomen due to lymphoma, it was determined that small bowel resection combined with primary anastomosis yields the best short-term results, along with the lowest morbidity and 30-day mortality rates. However, there are differences in lymphoma presentations, and most patients with BL often have an existing diagnosis, are receiving chemotherapy, and frequently present with bowel perforation [21].

Bowel obstruction can be the first symptom of BL-related surgical emergencies. If this is the case, bowel resection might be necessary, which can be supplemented by chemotherapy and radiation. When resection is not viable, chemotherapy and radiation can reduce the risk of recurrence. In extensive intraabdominal disease, surgery should be limited to a biopsy and ostomy [6].

A common cause of small bowel obstruction due to BL is intussusception, with surgical resection being the recommended management. Unlike in small children, 90% of intussusception in adolescents is caused by a well-established lead point, such as a carcinoma, a polyp, Meckel’s diverticulum, or benign tumors [22]. Although rare in children, malignant causes such as lymphomas and small bowel tumors also increase in incidence with age [23,24]. Yagmur et al. presented 17- and 19-year-old patients with terminal ileal BL-caused intussusception who required emergency surgery, bowel resection, and/or hemicolectomy with favorable outcomes [25]. Many case reports describe similar presentations managed with surgical resection and adjuvant chemotherapy, with diagnosis only confirmed postoperatively [19]. Interestingly, in our case, we could not establish a connection between the initial intussusception and the BL.

In our case, the surgical team opted for minimally invasive access to better approach the abdominal wall infiltrates, as well as due to the known advantages of MIS. Others have described similar port positioning to better expose the tumoral tissue based on imaging for the treatment of abdominal wall malignancies. Meshikes et al. used a partial thoracoabdominal position, exposing the left flank trocars inserted in the left lateral abdominal wall on the axillary lines for the laparoscopic excision of an abdominal desmoid tumor [26]. Previous studies have shown the high accuracy of laparoscopic biopsies for lymphoproliferative diseases and pediatric cancers [27,28] not detected through preoperative imaging [29].

Regarding biopsies, only a few publications exist in the literature about minimally invasive approaches for BL resections in children. Similarly to our case, Murakami et al. presented a successful laparoscopy-assisted radical resection of a large intraabdominal tumor in a 13-year-old boy. After right hemicolectomy and retroperitoneal lymph node resection, the tumor was removed via an umbilical incision. The intestine was reconstructed with a functional end-to-end anastomosis. The pathology report indicated a BL-like lymphoma with an 11q aberration [30].

Explorative laparoscopy seems to be a useful tool in managing surgical emergencies when the diagnosis is uncertain. Vrancx et al. reported the case of a 17-year-old patient with atypical right lower quadrant pain. An explorative laparoscopy was performed. No signs of acute inflammation were seen, nor fluid in the abdomen, and a large ileal mass was observed proximal to the ileocecal valve with multiple mesenteric adenopathies. An excision biopsy and histologic and immunohistochemical analyses confirmed the diagnosis [31]. Wang et al. reported a case of a 10-year-old boy who was diagnosed with acute appendicitis caused by ileocolic intussusception with appendiceal invagination, underwent a one-trocar laparoscopy, and received antibiotics as treatment. His symptoms recurred just ten days after being discharged. A subsequent colonoscopy revealed ileocecal BL as the pathological lead point [32]. Unforeseen occurrences can sometimes happen after a minimally invasive biopsy for BL. Metzelder et al. reported port-site metastasis resulting from a laparoscopic biopsy of post-transplant mesenteric BL [33].

## 4. Conclusions

Laparoscopy-assisted removal can be a useful approach for pediatric solid abdominal tumors with abdominal wall infiltrates, such as BL, causing intestinal obstruction. However, surgery may not represent the standard of care in BL. Nevertheless, the surgeon should carefully assess indications and procedures based on preoperative imaging and intraoperative findings in cases requiring emergency intervention. The role of ablative MIS in pediatric BL remains limited.

## 5. Limitations

We presented a clinical case report on managing BL in an adolescent using a minimally invasive approach. It is important to recognize the limitations and impact of case studies compared to more extensive cohorts.

## Figures and Tables

**Table 1:** Diagnostic immunohistochemical panel with antibody characteristics.

Antibodies	Clone	Manufacturer	Cat. No.
CD20	L26	Dako	M075501-2
PAX5	DAK-Pax5	Dako	GA65061-2
CD10	DAK-CD10	Dako	GA78661-2
BCL-2	124	Cell Marque	226M-95
BCL-6	PG-B6p	Dako	M721101-2
IgM	IgM	Dako	GA51361-2
CD23	DAK-CD23	Dako	GA78161-2
CD3	F7.2.38	Agilent Dako	M725401-2
Cyclina D1	EP12	Cell Marque	241R-46
TdT	TdT-339	Leica	50-255-1963
c-Myc	9E10	Santa Cruz	sc-40
EBV-LMP	CS 1-4	Dako	IR75361-2
Ki67	MIB-1	Dako	GA62661-2

Visualization reagent: EnVision FLEX/HRP (Agilent Dako, Santa Clara, CA, USA) in combination with 3,3′-diaminobenzidine tetrahydrochloride (DAB) chromogen; negative control: normal serum was substituted for the primary antibody; dilution: according to the manufacturer’s instructions. Antibodies manufacturer’s location: Dako—Santa Clara, CA, USA; Cell Marque—Rocklin, CA, USA; Leica—Nussloch, Germany; Santa Cruz—Dallas, TX, USA.

## Data Availability

The data generated in the present study are included in the figures and tables of this article.

## Abbreviations

BL	Burkitt’s lymphoma
B-NHL	B-cell non-Hodgkin’s lymphomas
CBC	complete blood count
CT	computer tomography
EBV	Epstein–Barr virus
ED	emergency department
FDG	fluorodeoxyglucose
LDH	lactate dehydrogenase
MIS	minimally invasive surgery
IGH-14q32	immunoglobulin heavy chain gene on chromosome 14q32
HGBL	High-grade B-cell lymphoma
NOS	not otherwise specified
MTX	methotrexate
PET	positron emission tomography
US	ultrasound
